# The CD40-CD154 axis in transplantation: from immunobiology to therapeutic targeting

**DOI:** 10.3389/fimmu.2026.1825653

**Published:** 2026-05-28

**Authors:** Kunxiong Wang, Tao Li, Ting Yan, Hidetaka Hara, Yi Wang

**Affiliations:** 1 Department of Kidney Transplantation, The Second Affiliated Hospital of Hainan Medical University, Haikou, China; 2 The Transplantation Institute of Hainan Medical University, Haikou, China

**Keywords:** CD154, CD40, CD40L, costimulation blockade, transplantation, xenotransplantation

## Abstract

The CD40-CD154 costimulatory axis serves as a central hub bridging innate and adaptive immunity, regulating antigen presentation, T-cell priming, B-cell activation, germinal center formation, and antibody class switching. In transplantation, this pathway drives donor-reactive T-cell expansion, dendritic cell (DC) licensing, and donor-specific antibody generation, contributing to both acute and chronic allograft rejection. Recent discoveries have expanded the classical linear model into a multidimensional signaling network. This network encompasses canonical CD40-TNF receptor-associated factor (TRAF)-NF-κB signaling, CD154 reverse signaling, and alternative receptor engagement—particularly through integrins such as CD11b. These insights explain the differential efficacy of CD154 versus CD40 blockade and inform next-generation therapeutic design. Concurrently, renewed clinical interest has emerged following the development of fragment crystallizable (Fc)-engineered anti-CD154 antibodies that circumvent first-generation thromboembolic toxicity. Here, we synthesize current understanding of CD40-CD154 molecular architecture, upstream triggers, downstream signaling networks, crosstalk with other immune pathways, and cell type-specific outputs. We evaluate therapeutic candidates in clinical development, discuss unresolved questions regarding long-term safety and biomarker development, and highlight future directions including cell-targeted intervention and tolerance-inducing combination strategies.

## 1 Introduction

The CD40–CD154 pathway is one of the best-characterized costimulatory axes in immunology and has emerged as a key therapeutic target in transplantation, autoimmunity, and cancer immunotherapy. Initially defined as an essential mediator of T cell-dependent B-cell help, this signaling system is now understood to regulate a broad spectrum of immune processes, including dendritic cell (DC) licensing, cytotoxic T-cell priming, inflammatory cytokine production, macrophage activation, and the balance between effector and regulatory immune responses. In transplantation, these diverse functions place the CD40–CD154 axis at the center of alloimmune activation.

Mechanistically, CD40–CD154 signaling cannot be viewed simply as a single ligand–receptor interaction. Instead, it functions within a broader regulatory network. This network is shaped by membrane organization, signal adaptor recruitment, receptor shedding, reverse signaling, and crosstalk with multiple immune pathways. In recent years, evidence has accumulated that CD154 can engage receptors beyond CD40, and that the biological consequences of pathway activation are highly dependent on cell lineage, tissue context, and inflammatory milieu. At the translational level, these mechanistic insights have driven renewed interest in therapeutic blockade of the pathway, particularly after the development of second-generation agents designed to reduce thromboembolic risk.

This review provides an updated synthesis of the structural and functional biology of CD40 and CD154, the multidimensional signaling network downstream of pathway activation, its integration with other immune circuits, and the current status of therapeutic targeting in transplantation and related fields.

## 2 Molecular structure, expression profile, and immunological significance of CD40 and CD154

### 2.1 Molecular structure and expression pattern of CD40 and CD154

CD40 is a transmembrane glycoprotein of the tumor necrosis factor receptor (TNFR) superfamily that is broadly expressed on professional antigen-presenting cells (APCs), including DCs, B cells, and macrophages, as well as on endothelial cells, epithelial cells, and certain non-immune cell populations (1). Its ligand, CD154, also known as CD40L, is predominantly expressed on activated CD4+ T cells, but can also be detected on platelets, natural killer cells, mast cells, and some malignant cells (1).

Although CD154 is predominantly expressed on activated CD4+ T cells and platelets, it can also be detected on antigen-presenting cells (APCs) such as macrophages and dendritic cells under inflammatory conditions. This broader expression pattern suggests potential autocrine or paracrine signaling loops that may amplify immune responses in transplantation (2).

Importantly, CD154 is not restricted to canonical CD40 binding. It can also interact with multiple integrins, including αIIbβ3, αMβ2, α5β1, αvβ3, and α4β1, thereby extending its biological impact to thrombosis, leukocyte adhesion, inflammatory amplification, and cell activation (3). Within the bone marrow microenvironment, CD154 has also been implicated in the regulation of early neutrophil development and migratory programming, suggesting a previously underappreciated role in myeloid ontogeny (4).

CD40 itself is subject to post-translational regulation. Its extracellular domain can be cleaved by a disintegrin and metalloproteinase 17 (ADAM17) to generate soluble CD40 (sCD40), which may function as a decoy receptor and has been proposed as a candidate biomarker reflecting chronic inflammatory activity (5).In parallel, CD154 itself is subject to ectodomain shedding by ADAM17 and ADAM10, generating soluble CD154 (sCD154) (6). Elevated sCD154 levels have been reported in allograft rejection, where it may act as a systemic agonist to activate CD40 on distant cells or interfere with therapeutic antibodies (7).

### 2.2 Central role of CD40–CD154 signaling in adaptive immunity

The CD40–CD154 axis is a critical bridge between innate and adaptive immunity. In T cell-dependent humoral responses, it is indispensable for B-cell activation, proliferation, germinal center formation, plasma cell differentiation, and immunoglobulin class-switch recombination (8). In parallel, CD40 signaling licenses DCs, enhances antigen presentation, sustains major histocompatibility complex (MHC) class I expression, and promotes effective CD8+ T-cell priming (9).

In transplantation, this pathway is a major driver of donor-reactive adaptive immunity. It promotes alloreactive T-cell activation and expansion, supports helper T-cell function, and facilitates donor-specific antibody production, thereby contributing to both acute cellular rejection and chronic antibody-mediated graft injury (10). Experimental models have shown that interruption of CD40–CD154 interactions suppresses donor-reactive T-cell frequencies, reduces graft infiltration, and promotes the expansion of regulatory T-cell populations, including Foxp3+ induced regulatory T cells (iTregs), with corresponding prolongation of graft survival (11). Notably, in nonhuman primate kidney transplantation models, anti-CD154 monoclonal antibody treatment has been associated with significantly longer rejection-free survival than anti-CD40 therapy, with reported median survival times of 352 days versus 131 days, respectively (12). These findings suggest that CD154 may mediate nonredundant immunological functions that are not fully recapitulated by CD40 blockade alone. Supporting this concept, CD154 also interacts with CD11b to induce interleukin-1β (IL-1β) production and inhibit iTreg generation, thereby introducing an additional layer of immune regulation beyond the canonical CD40 pathway (13).

In addition to its well-established expression on APCs, CD40 is also expressed on a subset of CD4+ T cells, termed Th40 cells. These cells are enriched among autoreactive effectors and exhibit a pro-inflammatory phenotype. While their role in transplantation has not been fully elucidated, they may contribute to allograft rejection by directly responding to CD154-expressing cells or by modulating local immune responses (14, 15).

### 2.3 Translational relevance of therapeutic targeting

Because of its central role in alloimmunity, the CD40–CD154 pathway has become a major target for strategies aimed at inducing transplant tolerance or reducing reliance on conventional broad immunosuppression. Early clinical efforts using anti-CD154 antibodies were curtailed by thromboembolic complications, largely attributed to Fc-dependent platelet activation (16). However, a new generation of humanized and Fc-engineered antibodies, such as TNX-1500 and related agents, has been designed to retain potent immunomodulatory activity while minimizing platelet activation and prothrombotic effects (17).

In xenotransplantation, blockade of the CD40–CD154 pathway has played a decisive role in extending graft survival in preclinical pig-to-primate models and has contributed to the immunological foundation underlying clinical pig organ transplantation efforts (18). Moreover, combination strategies appear particularly promising. In murine cardiac transplantation, sustained CD40L blockade combined with cytotoxic T-lymphocyte-associated protein 4 (CTLA4)-Ig has enabled long-term rejection-free graft survival without donor-specific antibody formation (19). Collectively, these advances support the view that precise modulation of CD40–CD154 signaling may not only complement but potentially replace some conventional immunosuppressive approaches in selected settings.

## 3 Comprehensive signaling landscape of the CD40–CD154 pathway

### 3.1 Upstream initiating events

#### 3.1.1 T-cell activation as the prerequisite for CD154 expression

**Figure 1:**
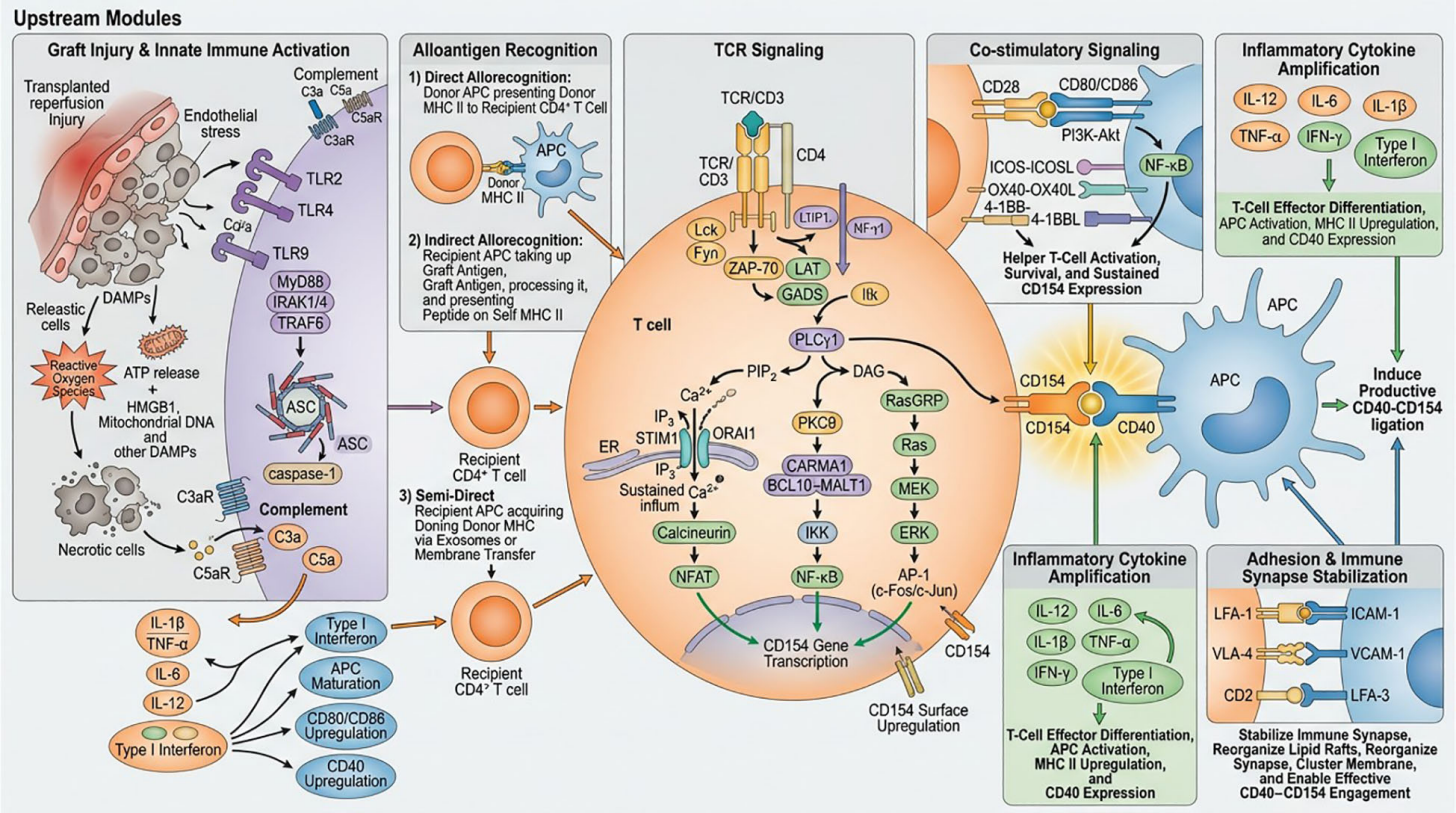
Upstream initiating events that drive CD40–CD154 activation in transplantation immunity. This schematic illustrates the coordinated upstream processes that culminate in productive CD40–CD154 ligation between activated CD4+ T cells and antigen-presenting cells (APCs) during transplantation. Graft injury, particularly ischemia–reperfusion injury, triggers the release of damage-associated molecular patterns (DAMPs), including ATP, HMGB1, and mitochondrial DNA.These DAMPs activate innate immune sensors such as TLR2, TLR4, TLR9, the MyD88-IRAK-TRAF6 axis, the NLRP3 inflammasome, and complement receptors C3aR/C5aR. These events promote APC maturation, inflammatory cytokine production, and upregulation of CD40 and co-stimulatory molecules. In parallel, donor alloantigens are recognized through direct, indirect, and semi-direct allorecognition pathways, leading to CD4+ T-cell activation. TCR/CD3 engagement with peptide–MHC II, together with CD4 co-receptor signaling, activates Lck, ZAP-70, LAT, SLP-76, and PLCγ1, which bifurcates into Ca2+/calcineurin/NFAT and DAG-dependent PKCθ/NF-κB and Ras–ERK/AP-1 pathways to induce CD154 transcription and surface expression. Co-stimulatory signals, dominated by CD28–CD80/CD86 and reinforced by ICOS, OX40, and 4-1BB pathways, further stabilize helper T-cell activation and sustain CD154 expression. Concurrently, inflammatory cytokines including IL-12, IL-6, IL-1β, TNF-α, IFN-γ, and type I interferons amplify APC activation, MHC II expression, and T-cell effector differentiation. Adhesion molecules such as LFA-1–ICAM-1, VLA-4–VCAM-1, and CD2–LFA-3 strengthen immune synapse formation and membrane clustering, enabling efficient CD40–CD154 engagement. As recently recognized, CD40 is also expressed on a subset of T cells (Th40 cells), and CD154 can be detected on APCs under inflammatory conditions; these non-canonical expression patterns are indicated in the figure. Together, these integrated upstream events establish the molecular conditions necessary for initiation of the CD40–CD154 signaling axis in alloimmunity.

CD154 is not expressed on resting T cells. Its induction depends on T-cell receptor (TCR) recognition of cognate antigen and subsequent T-cell activation (20). In transplantation, recipient CD4+ T cells recognize donor-derived alloantigens either directly on donor APCs or indirectly on recipient APCs presenting processed donor peptides. In the presence of coordinated TCR and CD28 costimulatory signaling, CD154 is rapidly upregulated within hours after T-cell activation, conferring the capacity to engage CD40 on APCs and B cells (21) (**Figure 1**).

#### 3.1.2 Lipid raft-dependent signaling platforms as a structural requirement

Effective CD40–CD154 signaling requires a specialized membrane microenvironment. Upon ligand engagement, CD40 undergoes oligomerization and is recruited into lipid rafts, where downstream adaptor proteins can be efficiently assembled (22). CD154 similarly depends on its transmembrane domain for localization to lipid rafts in activated T cells. Substitution of the CD154 transmembrane domain with that of transferrin receptor I prevents lipid raft recruitment and impairs activation of protein kinase B (Akt) and p38 mitogen-activated protein kinase (MAPK) pathways. This demonstrates that spatial membrane organization is essential for full signaling competence (23). Thus, lipid rafts function as highly ordered signaling platforms that enable productive TNF receptor-associated factor (TRAF) recruitment and NF-κB pathway activation.

#### 3.1.3 Triggering scenarios in transplantation

In transplantation, the CD40–CD154 pathway can be triggered in several coordinated settings: during direct donor–recipient APC–T-cell interactions, indirect alloantigen presentation by recipient APCs, and inflammatory activation within the graft microenvironment. Ischemia–reperfusion injury, innate immune activation, and vascular inflammation further amplify the likelihood of pathway engagement by increasing APC maturation, endothelial activation, and T-cell recruitment. In particular, ischemia–reperfusion injury has been shown to upregulate CD40 expression on graft endothelial cells and passenger leukocytes, thereby sensitizing the graft to CD40–CD154-mediated inflammation (24). These combined events create the immunological context in which CD40–CD154 signaling becomes a major amplifier of alloimmune injury.

### 3.2 Downstream signaling architecture: from a linear axis to a multidimensional network

**Figure 2:**
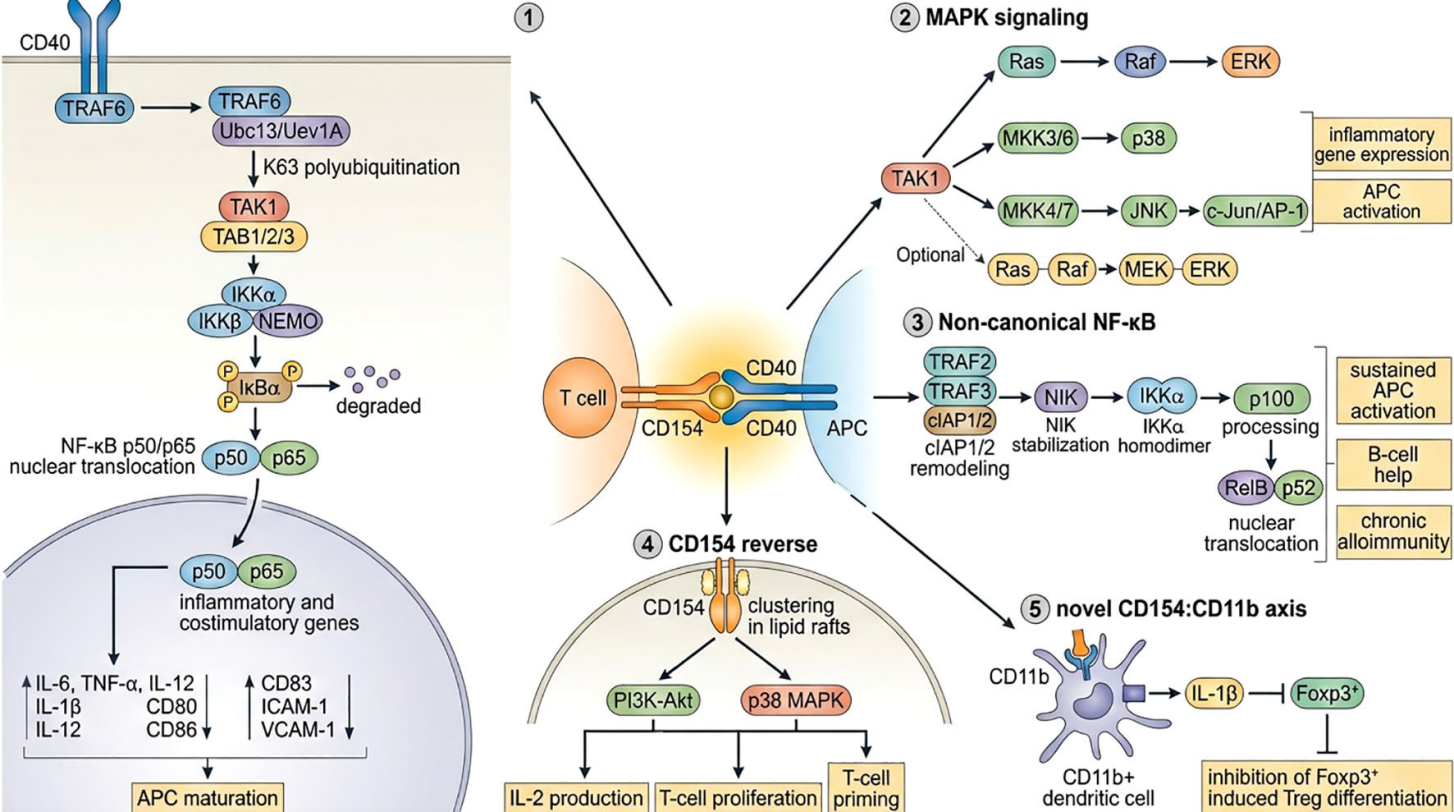
Downstream signaling network of the CD40–CD154 axis in transplantation immunity. Engagement of CD154 on activated CD4+ T cells with CD40 on antigen-presenting cells initiates a multilayered signaling network that amplifies immune responses. The classical CD40 pathway proceeds through TRAF6-dependent K63 polyubiquitination, TAK1–TAB complex activation, IKK-mediated IκBα degradation, and nuclear translocation of canonical NF-κB (p50/p65), resulting in the induction of inflammatory cytokines and co-stimulatory molecules. In parallel, TAK1 activates the p38 and JNK/AP-1 MAPK branches, further promoting inflammatory gene expression and APC activation, while TRAF2/TRAF3/cIAP remodeling stabilizes NIK to engage non-canonical NF-κB signaling via RelB/p52. On the T-cell side, CD154 clustering within lipid rafts mediates reverse signaling through PI3K–Akt and p38, enhancing IL-2 production, proliferation, and priming. In addition, the newly recognized CD154:CD11b axis in myeloid dendritic cells drives IL-1β release and suppresses Foxp3+ induced regulatory T-cell differentiation. Collectively, these interconnected pathways position CD40–CD154 signaling as a central hub linking APC licensing, effector T-cell activation, impaired regulation, and chronic alloimmune injury in transplantation.

The traditional model of CD40–CD154 signaling centered on the CD40–TRAF–NF-κB axis. While this framework remains fundamental, recent evidence indicates that the pathway is better understood as a multidimensional signaling network comprising at least three interconnected modules: canonical CD40 signaling, reverse and noncanonical signaling associated with CD154, and alternative receptor-mediated signaling involving integrins such as CD11b (**Figure 2**).

#### 3.2.1 Canonical CD40–TRAF signaling

Upon ligation, CD40 recruits TNF receptor-associated factors (TRAFs), especially TRAF2, TRAF3, TRAF5, and TRAF6, leading to activation of canonical and noncanonical NF-κB pathways, MAPK cascades, and phosphoinositide-dependent signaling modules (25).This dual activation is particularly critical in B cells, where both pathways cooperate to drive proliferation and differentiation (26).These events collectively regulate APC maturation, inflammatory cytokine production, B-cell survival, and class-switch recombination. In DCs, this signaling program promotes expression of costimulatory molecules and supports efficient T-cell priming. The essential role of CD40–CD154 signaling in T-cell priming and effector differentiation has been comprehensively reviewed elsewhere (27).

#### 3.2.2 Reverse and alternative receptor-mediated signaling by CD154

CD154 is increasingly recognized as a signaling-competent molecule rather than a passive ligand (28). Upon engagement, CD154 can initiate reverse signaling through pathways involving PI3K/Akt and p38 MAPK in T cells, modulating cytokine production and cell survival. Furthermore, CD154 interacts with multiple integrins including CD11b, α5β1, αvβ3, α4β1, and αIIbβ3. Among these, the CD154:CD11b axis has attracted particular attention in transplantation immunology: it promotes IL-1β production and constrains induced regulatory T cell (iTreg) generation, thereby shifting the balance toward effector immunity (29). Emerging evidence also suggests that CD154 reverse signaling may influence Th17 differentiation and memory T cell formation (30). These findings indicate that therapeutic targeting of CD154 may have broader immunological consequences than CD40 blockade alone, potentially affecting both adaptive and innate compartments. Further studies are needed to dissect the cell-type-specific outcomes of CD154 reverse signaling in allograft rejection and tolerance.

#### 3.2.3 Conceptual transition to a three-dimensional network

Taken together, current evidence supports a conceptual shift from a simple linear CD40–CD154 pathway to a three-dimensional signaling network integrating the canonical CD40–TRAF–NF-κB axis, CD154 reverse signaling, and the CD154:CD11b–IL-1β regulatory axis. This updated framework provides a more complete explanation for the differential effects observed between anti-CD40 and anti-CD154 therapies and may help guide the rational design of next-generation costimulation blockade strategies.

### 3.3 Cell- and tissue-specific signaling outputs

**Figure 3:**
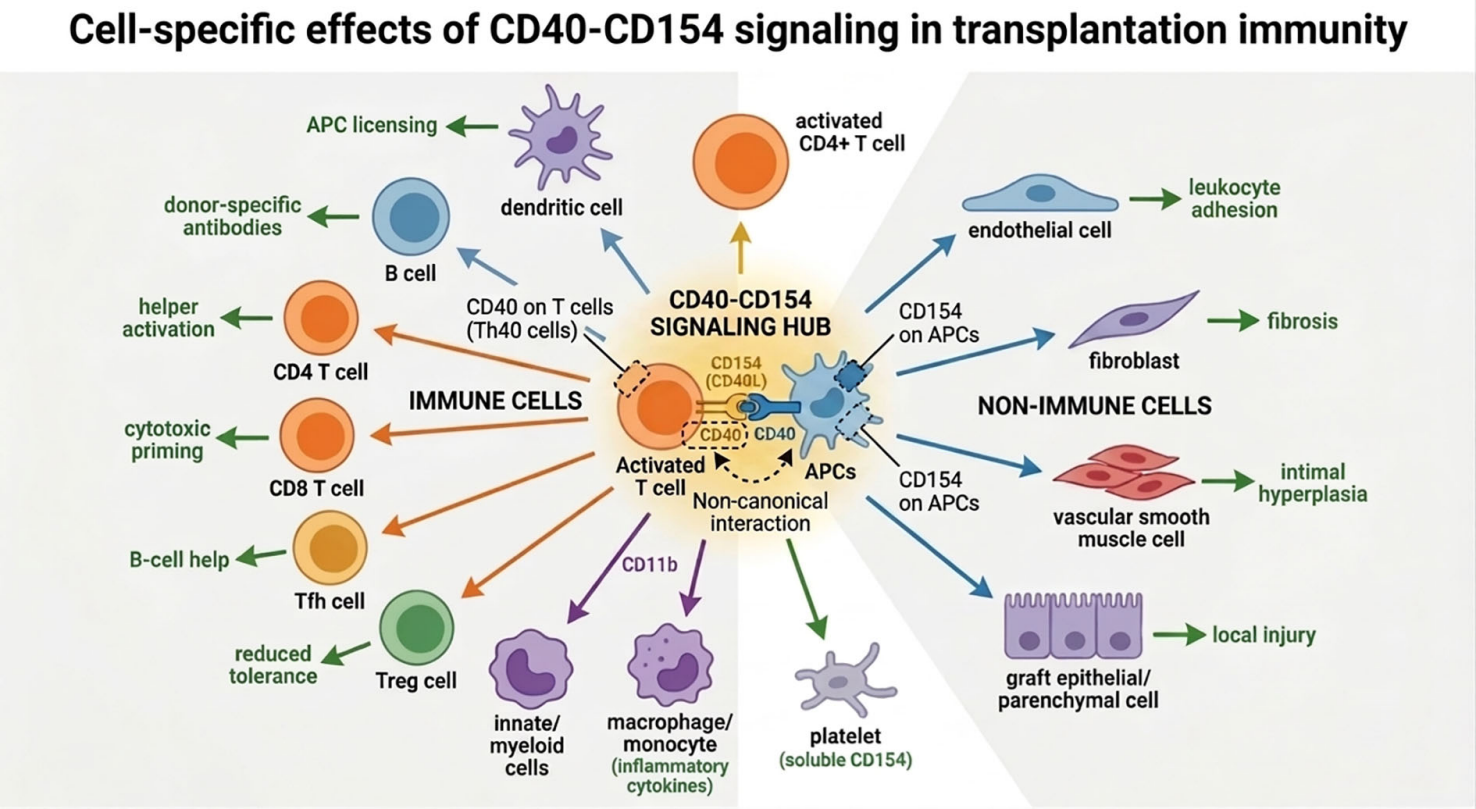
Cell-type-specific effects of CD40-CD154 signaling in transplantation immunity. This schematic summarizes the cell-type-specific functions of the CD40-CD154 pathway in transplantation. Activated CD4+ T cell-derived CD154 engages CD40 on multiple immune and graft-resident cell types, promoting dendritic cell licensing, B-cell activation and donor-specific antibody production, effector T-cell responses, macrophage inflammatory activation, platelet-derived soluble CD154 release, endothelial activation, fibrosis, vascular remodeling, and parenchymal injury, while weakening regulatory T-cell-associated tolerance. Consistent with recent findings, CD40 is now depicted on T cells (Th40 cells) to indicate their potential as direct effectors, and CD154 is shown on APCs to represent non-canonical expression. Collectively, these effects amplify graft inflammation and chronic allograft injury.

The biological consequences of CD40–CD154 signaling are highly context dependent. In B cells, the pathway drives proliferation, immunoglobulin class switching, and immunoglobulin E (IgE) generation, underscoring its central role in humoral immunity (31). In DCs, CD40 activation upregulates costimulatory molecules and antigen-presentation machinery, including MHC class I and II and intercellular adhesion molecule 1 (ICAM-1), and promotes IL-12 secretion, thereby favoring Th1-type immune responses (32) (**Figure 3**).

Cell-specific regulation is also evident in tolerance-associated pathways. A CD19+CD5+CD1d+ regulatory B-cell subset has been shown to prolong cardiac allograft survival by suppressing CD40–TRAF6 signaling in DCs, and this protective effect is lost when TRAF6 is selectively deleted in DCs, highlighting the importance of lineage-specific signaling architecture in transplant tolerance (33). At the T-cell level, CD154:CD11b interactions inhibit forkhead box protein P3 (Foxp3+) iTreg generation by promoting IL-1β release, whereas blockade of this axis increases iTreg frequency and prolongs graft survival (13).

Beyond classical immune cells, CD40 is also expressed on non-hematopoietic cell types that may influence transplant outcomes. In pancreatic islet transplantation, CD40 expression on beta cells has been implicated in inflammatory destruction during type 1 diabetes and may render islet grafts vulnerable to CD154-mediated injury (34). Similarly, CD40 is present on neurons, astrocytes, and microglia, where it contributes to neuroinflammation. These tissue-specific expression patterns may be particularly relevant when considering islet or neuronal transplants in autoimmune or inflammatory settings (35, 36).

In endothelial and Müller cells, CD40 expression can be modulated by advanced glycation end products (AGEs), leading to upregulation of inflammatory mediators such as C-C motif chemokine ligand 2 (CCL2) and ICAM-1 and contributing to the pathogenesis of diabetic retinopathy (37). In cancer immunotherapy, tumor-targeted bispecific constructs such as fibroblast activation protein (FAP)-CD40 have been developed to selectively activate CD40 signaling within the tumor microenvironment, enhancing local antigen presentation and T-cell responses while limiting systemic toxicity (38).

Overall, these observations indicate that the CD40–CD154 pathway does not elicit a uniform biological program. Rather, it generates highly cell- and tissue-specific outputs that shape immunity, tolerance, and disease progression in distinct ways.

## 4 Core crosstalk with BCR, TLR, BAFF/BLyS, and other molecular pathways

### 4.1 Synergy with B-cell receptor signaling

B-cell receptor (BCR) signaling and CD40 signaling cooperate at multiple stages of B-cell development and activation. In naive B cells, BCR engagement may induce apoptosis unless timely CD40-dependent NF-κB activation provides the survival signals needed to rescue activated cells, indicating that these pathways are integrated in a finely tuned, non-monotonic manner during positive and negative selection (39). In germinal centers, CD40 and BCR signals converge with T follicular helper-derived interleukin-21 (IL-21) to induce c-MYC and phospho-S6, thereby promoting positive selection and B-cell fate commitment (40).

Mechanobiological regulation has also emerged as an important component of this crosstalk. CD40–CD154 catch-bond formation is required for efficient cooperation between BCR and CD40 signaling, and CD40L mutations associated with X-linked hyper-IgM syndrome impair this mechanical interaction and weaken class-switch recombination (41). In chronic lymphocytic leukemia (CLL), BCR signaling suppresses miR-29 expression, resulting in TRAF4 upregulation and amplified CD40–NF-κB signaling. This establishes a miR-29-TRAF4-CD40 signaling axis that can be disrupted by Bruton’s tyrosine kinase (BTK) inhibition with ibrutinib (42). Additional work has shown that in TANK-binding kinase 1 (TBK1)-deficient B cells, combined BCR and CD40 stimulation increases interferon regulatory factor 4 (IRF4) while suppressing B-cell lymphoma 6 (BCL6), impairing germinal center formation and durable memory humoral responses (43).

### 4.2 Crosstalk with Toll-like receptor pathways

CD40–CD154 signaling intersects extensively with Toll-like receptor (TLR) pathways. In systemic lupus erythematosus, memory B cells are relatively hyporesponsive to BCR engagement but retain sensitivity to TLR signaling, particularly through TLR7/8, as well as to type I interferons and CD40–CD154 stimulation (44). In osteoarthritis, B cells exposed to combined TLR-dependent and CD40–BCR activation acquire a more mature phenotype, display accelerated differentiation, and show enhanced antibody secretion, suggesting that these pathways cooperate in local autoimmune inflammation (45).

Pathogen-associated ligands can also synchronize BCR and TLR activation. Multivalent antigens or polymeric lipopolysaccharides can concurrently crosslink BCRs and TLRs, synergistically promoting B-cell proliferation and class-switch recombination within defined concentration windows (46). In CLL, TLR9 activation increases CD40 expression and promotes translation of pro-survival proteins, whereas ibrutinib suppresses this effect and thereby attenuates CD40-mediated resistance to venetoclax (47).

### 4.3 Crosstalk with BAFF/BLyS

B-cell activating factor (BAFF), also known as B-lymphocyte stimulator (BLyS), functions in concert with CD40–CD154 signaling to regulate B-cell survival, maturation, and alloimmune responses. In chronic graft-versus-host disease (cGVHD) models following allogeneic bone marrow transplantation, elevated BAFF levels are associated with persistent B cells activated through the BCR. BAFF enhances BCR responsiveness by upregulating NOTCH2 and SYK, thereby aggravating cGVHD manifestations, expanding circulating GL7+B cells, and promoting alloantibody production. These findings suggest that BAFF-mediated BCR potentiation may cooperate with CD40–CD154 signaling in pathogenic B-cell responses (48).

In individuals carrying heterozygous loss-of-function mutations in genes related to the CD40–CD154 pathway, IgD-negative naive B cells exhibit reduced BAFF receptor (BAFF-R) expression and diminished responsiveness to BAFF, BCR, and CD40 stimulation, indicating that BAFF-R and CD40 signaling jointly participate in early B-cell selection and survival (49). Mature B cells coexpress BCR, TLRs, CD40, and BAFF-R, and these receptors form a complex signaling network that coordinates B-cell development, differentiation, survival, and antibody production. Within this network, CD40–CD154 remains a core driver of class-switch recombination and acts synergistically with BAFF-R-mediated survival pathways (50).

### 4.4 Crosstalk with additional molecular pathways

#### 4.4.1 Integrins and alternative CD154 receptors

CD154 can bind multiple integrins beyond CD40, including αIIbβ3 (GPIIb/IIIa), αMβ2 (CD11b/CD18), α5β1 (VLA-5), αvβ3 (vitronectin receptor), and α4β1 (VLA-4). These interactions mediate diverse functions: platelet aggregation and thrombosis (αIIbβ3), leukocyte adhesion and transendothelial migration (α4β1, αMβ2), inflammatory amplification (αMβ2), and cell activation (α5β1, αvβ3). Thus, CD154 acts as a multifunctional ligand that extends its biological impact well beyond its canonical CD40-dependent effects. These alternative receptor engagements may contribute to transplant outcomes, inflammatory activation, and tolerance-related mechanisms (11, 29).

#### 4.4.2 RANK–RANKL

CD40 and receptor activator of nuclear factor-κB (RANK) are both members of the TNFR superfamily and share TRAF6 as a downstream signaling node. RANK ligand (RANKL) promotes DC survival, and because activated DCs may express RANKL, reciprocal DC–DC communication through either CD40L–CD40 or RANKL–RANK may shape DC survival and function (51).

#### 4.4.3 TNF-α

CD40 activation induces TNF-α production, and TNF-α in turn upregulates CD40 expression, forming a positive feedback loop that amplifies inflammatory responses. In cancer immunotherapy, TNF blockade may mitigate some of the toxicities associated with CD40 agonists (52).

#### 4.4.4 Fas/FasL

CD40 signaling modulates susceptibility to Fas/Fas ligand (FasL)-mediated apoptosis in a cell type -dependent manner. In DCs, TNF-related activation-induced cytokine (TRANCE, also known as RANKL) can counteract FasL-induced apoptosis, supporting immune homeostasis and regulating activation-induced cell death (51, 53).However, the outcome of CD40-Fas crosstalk is not uniform across all settings. In contrast to the pro-apoptotic effect reported in some cell types, engagement of CD40 and Fas in autoimmune-prone mouse models does not result in cell death but rather alters the homeostatic balance between autoaggressive (CD4+CD40+) and regulatory (CD4+CD25+FoxP3+) T cells (15). This observation may be particularly relevant for transplant recipients with underlying autoimmune diseases, such as type 1 diabetes or systemic lupus erythematosus, where pre-existing immune dysregulation could modify the response to CD40-targeted therapy. Further studies are required to define how CD40-Fas crosstalk influences allograft outcomes in the setting of autoimmunity.

#### 4.4.5 NF-κB-inducing kinase

Both CD40 and TLR pathways can activate NF-κB-inducing kinase (NIK), thereby engaging the noncanonical NF-κB2 pathway. NIK is required for CD40-mediated cross-presentation and therefore plays an important role in DC maturation and function (54).

#### 4.4.6 SOCS family proteins

CD40 signaling induces suppressor of cytokine signaling 3 (SOCS3) expression, rendering cells less responsive to subsequent TLR stimulation and establishing a state of cross-tolerance. Conversely, prior TLR activation can suppress subsequent CD40 responsiveness. This reciprocal regulation helps prevent excessive inflammation but may also be exploited during pathogen immune evasion (55).

#### 4.4.7 IRAK family proteins

CD40 stimulation downregulates interleukin-1 receptor-associated kinase (IRAK) expression while increasing the negative regulator IRAK-M (a negative regulator of TLR signaling), thereby suppressing TLR signaling and providing a molecular basis for cross-tolerance (55).

#### 4.4.8 ADAM17/TACE

ADAM17-mediated ectodomain shedding of CD40 generates soluble CD40, which may function as a decoy receptor that neutralizes CD154 and dampens inflammation. ADAM17 also cleaves other inflammatory mediators such as TNF-α and ICAM-1, emphasizing its role as a negative feedback regulator. Soluble CD40 may therefore serve as a biomarker of chronic inflammatory activity (5).

Similarly, ADAM10 and ADAM17 also cleave CD154 to produce soluble CD154 (sCD154), which can activate CD40 signaling in a paracrine manner and may serve as a biomarker of transplant rejection or autoimmune activity (6).

#### 4.4.9 Nitric oxide and peroxynitrite

Peroxynitrite can nitrate CD40 tyrosine residues, leading to receptor internalization and 20S proteasome-mediated degradation. Shear stress-dependent nitric oxide release may influence CD40 stability and contribute to vascular bed-specific patterns of CD40 expression, with potential relevance to atherosclerosis-prone regions (56).

#### 4.4.10 C4-binding protein

C4bp has been proposed as a second ligand for CD40, potentially linking complement activation to CD40 signaling and thereby bridging innate and adaptive immunity (57).

#### 4.4.11 Immune synapse polarity

T-cell polarity, especially microtubule cytoskeletal polarization, is a prerequisite for enrichment of CD154 at the immune synapse. Disruption of T-cell polarity abolishes CD154-dependent IL-12 secretion by DCs, highlighting the structural basis required for effective signal transmission through the CD40–CD154 axis (58).

## 5 Therapeutic targeting of the CD40–CD154 axis

Therapeutic targeting of the CD40–CD154 axis has emerged as one of the most compelling strategies for controlling allo- and xenoimmune responses. By interrupting a central costimulatory pathway linking T-cell help, antigen-presenting cell activation, germinal center formation, and antibody production, CD40/CD154 blockade can suppress both cellular and humoral rejection while also attenuating innate inflammatory amplification. These properties are particularly relevant to xenotransplantation, where adaptive immunity, endothelial activation, and thromboinflammatory injury coexist.

Initial enthusiasm focused on CD154 blockade, which showed striking efficacy in preclinical transplantation models. However, clinical development of first-generation anti-CD154 antibodies was halted by thromboembolic complications (16), largely attributed to Fc-mediated interactions with platelet-associated CD154. This safety signal shifted the field toward CD40-targeted agents, several of which have since entered clinical transplantation. Nonetheless, the target biology of CD154 remains highly attractive, especially because CD154 blockade may inhibit not only CD40 forward signaling but also broader CD154-dependent immune and platelet-associated effector functions. An overview of representative therapeutic agents targeting the CD40-CD154 pathway, including their development status and relevance to transplantation, is provided in **Table 1**.

**Table 1:** Representative therapeutic agents targeting the CD40–CD154 pathway.

Drug (Generic name/code)	Target	Class	Stage	Primary indication (s)	Investigated in transplantation?
Approved (none for CD40/CD154 in transplantation)					
None	–	–	–	–	–
Phase 3					
Dapirolizumab pegol (DZP)	CD154	Antibody (PEGylated)	Phase 3	SLE	No
Dazodalibep (VIB4920/HZN-4920)	CD154	Fusion protein	Phase 3	Sjögren’s syndrome	No
Phase 2/2a					
Frexalimab (SAR441344)	CD154	Antibody	Phase 2	MS, Sjögren’s	No
Tegoprubart (AT-1501)	CD154	Antibody (Fc-engineered)	Phase 2/3	ALS; NHP & human xenotransplantation	Yes (NHP kidney/islet; human xeno)
KPL-404	CD40	Antibody	Phase 2	Rheumatoid arthritis	No
Phase 1/1b					
IBI355	CD154	Antibody	Phase 1	Autoimmune diseases	No
BMS-986 (anti-CD40L domain antibody)	CD154	Antibody	Phase 2 (completed)	ITP	No
CD40-targeting peptides (e.g., OPT101)	CD40	Peptide	Phase 1	Type 1 diabetes	Not yet (proposed)
Discontinued/historical					
BG9588 (first-gen anti-CD154)	CD154	Antibody	Discontinued (thromboembolic toxicity)	SLE, renal transplant	Yes (historical human trials)
TNX-1500	CD154	Antibody (Fc-engineered)	Discontinued (development halted)	Autoimmunity; NHP transplantation	Preclinical only; no longer in human trials
Iscalimab (CFZ533)	CD40	Antibody	Discontinued (CIRRUS-1 failed)	Type 1 diabetes, lupus nephritis, Sjögren’s, kidney transplant	No longer in development
Bleselumab (ASKP1240)	CD40	Antibody	Discontinued (2021)	Prevention of kidney transplant rejection	Historical (human kidney, phase 1b/2) – now halted
Preclinical/investigational					
Small molecule inhibitors (e.g., from Chuang et al.)	CD40–CD154 interaction	Small molecule	Preclinical	Islet transplantation, T1D prevention	Yes (murine islet)
Next-generation anti-CD40L antibodies (e.g., KJ047)	CD154	Antibody (Fc-engineered, protease-resistant)	Preclinical	Transplant immunosuppression	Proposed (designed for combined use with IgG proteases)

SLE, systemic lupus erythematosus; MS, multiple sclerosis; ALS, amyotrophic lateral sclerosis; ITP, immune thrombocytopenia; T1D, type 1 diabetes; NHP, nonhuman primate.

Recent studies have renewed interest in this pathway from both mechanistic and translational perspectives. In nonhuman primates, AT-1501, a next-generation anti-CD40L monoclonal antibody, prolonged islet and kidney allograft survival and preserved graft function, supporting the feasibility of safer CD154-directed immunosuppression (59). In parallel, the expanding oncology literature has reinforced the concept that CD40 is a major immunologic hub whose effects are shaped by tissue context, inflammatory milieu, and combinatorial pathway modulation (60, 61). Although these studies are not transplantation-specific, they underscore the need to optimize both efficacy and tolerability when manipulating this axis. Currently, the anti-CD154 antibodies TNX-1500 and AT-1501 (tegoprubart) are the leading candidates in clinical development for transplantation. Among anti-CD40 antibodies, iscalimab (CFZ533) has shown promise in preclinical and early-phase studies, although bleselumab (ASKP1240) has been discontinued. All remain investigational; none have received regulatory approval for transplant immunosuppression.

Recent clinical experience has demonstrated the feasibility of tegoprubart-based immunosuppression in human xenotransplantation. In September 2023, the University of Maryland transplanted a 10-gene-edited pig heart into a 58-year-old man with end-stage heart failure using a tegoprubart-containing costimulation blockade regimen (62). Although the graft functioned initially, it developed progressive diastolic failure with evidence of antibody-mediated injury. Subsequent expanded-access pig kidney xenotransplants at Massachusetts General Hospital in collaboration with eGenesis (2024–2025) also incorporated tegoprubart as part of the immunosuppressive backbone. FDA has cleared multi-patient trials for xenotransplantation, including eGenesis’ EGEN-2784 and United Therapeutics’ UKidney program (IND cleared February 2025). These advances underscore the clinical momentum of CD40/CD154-targeted therapies in xenotransplantation.

The translational relevance of CD40/CD154 blockade is even more pronounced in xenotransplantation. In a framework analysis of candidate regimens for first-in-human pig organ transplantation, Bikhet et al. identified blockade of the CD40–CD154 pathway as a likely backbone of clinical immunosuppression (63). This view is consistent with recent pig-to-human and pig-to-baboon studies, in which successful xenograft support has relied on the integration of donor gene editing with potent costimulation blockade (64, 65). In this setting, targeting the CD40–CD154 axis helps restrain T-cell priming, xenoantibody generation, and myeloid-endothelial activation, thereby addressing several major barriers simultaneously.

Beyond monoclonal antibodies, small-molecule inhibitors of CD40–CD40L interaction are now entering the field. In preclinical models, these compounds improved pancreatic islet transplantation outcomes and prevented autoimmune diabetes, suggesting a potential future role as oral adjuncts or maintenance-phase agents (66). Although still early in development, such platforms may broaden the therapeutic landscape and facilitate more flexible pathway inhibition.

From a translational perspective, the most promising next-generation strategies for transplantation include Fc-engineered anti-CD154 antibodies (e.g., TNX-1500) that avoid thromboembolic toxicity, as well as combination approaches that pair CD40/CD154 blockade with CTLA4-Ig, anti-CD28, or regulatory cell therapies. Selective targeting of the CD154:CD11b axis may also offer a pathway to inhibit alloimmunity while preserving protective immunity.

Taken together, current evidence supports a shift from viewing the CD40–CD154 axis as a conventional T-cell costimulatory target to recognizing it as a broader immunoinflammatory checkpoint. In allotransplantation, this has driven sustained clinical development of anti-CD40 antibodies; in xenotransplantation, it has positioned CD40/CD154 blockade as a foundational component of next-generation regimens. The next phase of the field will likely depend on biomarker-guided patient selection, rational combination strategies, and the clinical re-entry of Fc-silenced anti-CD154 therapeutics.

## 6 Biomarkers and clinical monitoring

The development of reliable biomarkers is a prerequisite for precision targeting of the CD40–CD154 pathway. Soluble CD40, generated through ADAM17-dependent shedding, has emerged as a candidate marker of chronic inflammation and pathway activation (5). At the cellular level, suppression of CD40–TRAF6 signaling in DCs has been linked to Breg-mediated tolerance in transplantation models, suggesting that intracellular pathway states may provide mechanistic biomarkers of tolerance induction (33).

CD154 itself may also be clinically informative. Rapid induction of CD154 on antigen-specific CD4+ T cells has been associated with pathogenic immune activity and autoantibody titers, indicating potential utility in identifying disease-relevant T-cell populations (67). In interventional studies, tegoprubart has been shown to reduce a broad panel of inflammatory mediators in a dose-dependent manner, supporting its use for pharmacodynamic monitoring (68). Likewise, treatment with iscalimab in type 1 diabetes has been associated with attenuation of β-cell functional decline and improved glycated hemoglobin (HbA1c), suggesting that functional clinical endpoints may correlate with pathway inhibition (69).

Despite these advances, validated dynamic biomarker panels that can guide individualized treatment selection, dose adjustment, or safe tapering remain unavailable. Future biomarker programs should integrate soluble mediators, antigen-specific lymphocyte phenotyping, intracellular phospho-signaling, transcriptomics, and possibly spatial tissue profiling to build predictive models of response, tolerance, and infection risk.

## 7 Unresolved questions and future perspectives

### 7.1 Cell type-specific signaling remains incompletely defined

One of the major unresolved issues in the field is how CD40–CD154 signaling generates distinct outputs in different cell types. The same receptor–ligand pair can drive immunogenic, inflammatory, or tolerogenic programs depending on cellular context, receptor density, membrane organization, adaptor usage, and the surrounding cytokine milieu. A more granular understanding of these variables will be essential for the design of more selective therapeutic strategies.

### 7.2 Xenotransplantation-specific biology

In xenotransplantation, CD40–CD154 signaling appears to remain critically important, but its role may be modified by species-specific molecular incompatibilities. Long-term porcine islet xenograft survival has been achieved under CD40–CD154 blockade, and Fc-engineered anti-CD154 antibodies have reduced thrombotic complications in this setting (70). In gene-modified pig-to-baboon kidney transplantation, blockade-based immunosuppression controlled adaptive immunity effectively, although endocrine incompatibilities such as dysregulated renin–angiotensin signaling emerged as additional barriers (71). Of note, one study reported successful long-term survival of porcine kidney xenografts in nonhuman primates using only conventional immunosuppression without CD40/CD154 blockade, suggesting that the requirement for this pathway may be context-dependent (72).These observations indicate that xenotransplant success depends not only on suppressing rejection but also on understanding cross-species physiological mismatches. The apparent difference between the superiority of anti-CD154 over anti-CD40 in NHP allotransplantation (Section 2.2) and the successful long-term xenograft survival without CD40/CD154 blockade reflects context-dependent pathway engagement. In allotransplantation, the strong adaptive response to donor MHC antigens involves full CD40-CD154 signaling, whereas in xenotransplantation, multiple species-specific molecular incompatibilities (e.g., complement dysregulation, coagulation pathway mismatches) can modify the requirement for costimulation blockade. Thus, the necessity of CD40/CD154 targeting for graft survival depends critically on the transplant model and recipient species.

### 7.3 Safety, host defense, and pathway selectivity

Whether long-term pathway inhibition compromises antimicrobial immunity remains an important concern. Selective targeting of specific interactions, such as CD154:CD11b, may offer a way to suppress alloimmunity while preserving broader host defense capacity and memory T-cell formation (73). Future therapeutic development should therefore prioritize selective blockade, optimized antibody engineering, and tissue-targeted delivery systems.

### 7.4 Toward biomarker-guided combination immunotherapy

The next phase of translational development will likely depend on moving from single-agent blockade to biomarker-guided, mechanism-informed combination therapy. The most successful regimens may integrate costimulation blockade with coinhibitory pathway engagement, regulatory cell expansion, or metabolic control to reshape the immune system toward durable tolerance rather than transient suppression.

### 7.5 Small peptide-based CD40 targeting

Beyond monoclonal antibodies and small molecules, direct CD40-targeting peptides have been developed and shown to prevent autoimmune diabetes in preclinical models. These peptides disrupt the balance between CD4+CD40+ effector T cells and regulatory T cells, thereby restoring immune homeostasis (74). Such approaches offer high specificity and a potentially favorable safety profile. For transplantation, especially in recipients with autoimmune diseases, CD40-targeting peptides could be explored as adjunctive or tolerance-promoting agents.

### 7.6 The CD40-CD154 axis in vascularized composite allotransplantation

Vascularized composite allotransplantation (VCA), including hand, face, uterus, and limb transplantation, presents unique immunological challenges distinct from solid organ transplantation. VCA grafts contain skin and mucosa—highly immunogenic tissues that trigger robust alloimmune responses. Nearly all reported VCA recipients experience at least one acute rejection episode, and chronic rejection remains a significant barrier. Moreover, given that VCA is a non-life-saving procedure, the toxicity of conventional calcineurin inhibitor (CNI)-based regimens poses particular concerns, creating a strong rationale for CNI-sparing costimulation blockade strategies.

Belatacept, a CTLA4-Ig fusion protein blocking CD28–CD80/86 costimulation, has been successfully used in clinical hand transplantation as a CNI-free regimen (75). However, the CD40-CD154 axis may offer additional advantages in VCA given its critical role in T cell activation, germinal center formation, and antibody production. Preclinical studies in nonhuman primate VCA models have demonstrated that targeting CD40-CD154 signaling promotes long-term graft survival and reduces donor-specific antibody formation (76). The high antigenic burden of VCA grafts, coupled with their frequent acute rejection episodes and the life-altering consequences of graft loss, makes VCA an attractive setting for exploring costimulation blockade–based immunosuppression. Future clinical trials evaluating anti-CD154 or anti-CD40 antibodies in VCA recipients are warranted, particularly in combination with belatacept or regulatory cell therapies.

## 8 Conclusion

The CD40–CD154 signaling axis is a central regulator of transplantation immunity and a key mechanistic node connecting antigen presentation, T-cell activation, B-cell differentiation, antibody production, and immune regulation. Its functions extend beyond the classical CD40–CD154 interaction to include noncanonical receptor usage, reverse signaling, and extensive crosstalk with BCR, TLR, BAFF/BLyS, and multiple inflammatory pathways. These features help explain both the power and the complexity of targeting this axis in transplantation.

Preclinical and early clinical studies strongly support the therapeutic potential of CD40/CD154-directed intervention, particularly with newer agents designed to overcome the thrombotic limitations of first-generation anti-CD154 antibodies. At the same time, the field is moving toward a more nuanced view in which efficacy depends not merely on pathway blockade, but on understanding cell-specific signaling outputs, selecting rational combination partners, and incorporating biomarker-guided clinical monitoring.

Taken together, current evidence supports the CD40–CD154 pathway as both a mechanistic hub of alloimmunity and a promising gateway toward safer, more precise, and potentially tolerance-inducing immunotherapy. Continued advances in antibody engineering, systems immunology, and translational biomarker development are likely to determine how rapidly this promise can be realized in clinical transplantation.
